# Predictors of job satisfaction and burnout among tuberculosis management nurses and physicians

**DOI:** 10.4178/epih.e2016008

**Published:** 2016-03-09

**Authors:** Hae-Suk Seo, Hyunjoong Kim, Se-Min Hwang, Soo Hyun Hong, In-Young Lee

**Affiliations:** 1 Department of Tuberculosis, Seobuk Hospital, Seoul, Korea; 2 Department of Preventive Medicine, Korea University College of Medicine, Seoul, Korea; 3 Department of Epidemiology and Medical Informatics, Public Health Graduate School, Korea University, Seoul, Korea; 4 Gangdong Health Center, Seoul, Korea; 5 Korea Human Resource Development Institute for Health and Welfare, Cheongju, Korea; 6 Department of Food and Nutrition, Sookmyung Women’s University, Seoul, Korea; 7 Gangbuk Health Center, Seoul, Korea

**Keywords:** Burnout, Nurse, Stress, Tuberculosis, Physicians

## Abstract

**OBJECTIVES:**

This study examined job satisfaction, empowerment, job stress, and burnout among tuberculosis management nurses and physicians in public healthcare institutions.

**METHODS:**

This was a cross-sectional study analyzing survey data collected from 249 nurses and 57 physicians in 105 public health centers, three public tuberculosis hospitals, and one tertiary hospital. The survey questionnaire comprised general characteristics, work-related characteristics, and four index scales (job satisfaction, empowerment, job stress, and burnout). The two-sample *t*-test was used to estimate the mean differences in the four index scales. Multiple regression analysis was used to determine whether general and work-related characteristics affected the four index scales.

**RESULTS:**

The job satisfaction and empowerment scores of the nurses were lower than those of the physicians. Except for the tuberculosis-specialized hospitals alone, the average job satisfaction scores of nurses were higher than those of physicians. Moreover, the nurses reported more job stress and burnout than did the physicians in tuberculosis departments in public healthcare institutions in Korea; in particular, the burnout reported by nurses was significantly higher than that reported by physicians at the National Medical Center. Marital status, nursing position, number of coworkers, the average number of days of overtime work per month, self-rated health, and hospital type were associated with the four index scales.

**CONCLUSIONS:**

Overall, nurses were more vulnerable to job stress and burnout than physicians. Reducing the workload of nurses by ensuring the presence of sufficient nursing staff and equipment, as well as by equipping facilities to prevent tuberculosis infections, should be considered priorities.

## INTRODUCTION

In 2012, 8.6 million new tuberculosis (TB) cases and 1.3 million TB deaths were documented worldwide, demonstrating that TB remains a global health problem [1]. South Korea (hereafter Korea) had the highest incidence rate and highest mortality rate of TB among the Organization for Economic Cooperation and Development (OECD) countries in 2005. In Korea, the annual incidence rate of new TB infections was 87 per 100,000 population, which was well above the OECD average of 17.7; moreover, the mortality rate of TB in Korea of 10 per 100,000 population was five times greater than the OECD average of 2.1 [2].

In this context, TB can be seen as negatively impacting the national status of Korea, which has led to the adoption of a rigorous national strategy to resolve TB problems within a short time frame. In 2010, the Korean government launched the New 2020 Plan to eliminate TB in Korea. The major goal of this plan is the reduction of the incidence and mortality of TB to levels that are equivalent to those observed in other developed countries. In order to achieve these goals, the government implemented action plans, such as the expansion of the private-public mix (PPM), directly observed treatment, short-course drug therapy, and the establishment of a TB management system for TB-vulnerable groups, including the homeless [3].

Accordingly, a TB policy paradigm shift resulted in an increase of administrative tasks for TB management nurses in public health centers, as public health centers have been designated as the gateways for the administrative procedures required in the new national TB strategy. As part of the new strategy, public TB hospitals also became the priority hospitals for multi-drug-resistant (MDR) TB patients. The nurses working at these hospitals are therefore under severe stress due to concerns that they could become infected with TB.

Many studies regarding job satisfaction, stress, and work-related burnout have been conducted and subsequently utilized as evidence regarding effective forms of policy enforcement [4-13].

Few studies have not only evaluated the job satisfaction and stress of nurses who are in charge of TB patients, but also compared these variables among nurses with the corresponding results observed among physicians. Therefore, the purpose of this study was to analyze job satisfaction, empowerment, job stress, and burnout among TB management nurses in comparison with physicians in public healthcare institutions. This study also provides primary data for discussing solutions and macroscopic policy choices that seek to relieve the work-related stress of TB management nurses and improve their job satisfaction.

## MATERIALS AND METHODS

### Research subjects

The research subjects were distributed across 105 public health centers, three national public TB hospitals (Seoul Seobuk Hospital, Masan National Hospital, and Mokpo National Hospital), and one tertiary hospital (National Medical Center). The research subjects comprised 249 nurses and 57 physicians who responded to the survey (response rate=91.0%) and were involved in diagnosing and treating TB at these institutions.

### Research procedure and content

Our research was conducted over five months, from August to December 2013. The questionnaire was composed of questions assessing the subjects’ general characteristics, work-related characteristics, and four index scales (job satisfaction, empowerment, job stress, and burnout). The general characteristics included sex, age, marital status, educational level, monthly family income, and self-rated health. An income grade was determined by the total monthly household income level using a household income table from 2012 [14]. The work-related characteristics consisted of institution type, job type, job position, number of coworkers, years in the TB department, and the average number of days of overtime work per month.

In order to measure job satisfaction, this study utilized a form of the Minnesota Satisfaction Questionnaire tool that was modified, revised, and implemented by Lee & Park [15], with a Cronbach’s alpha of 0.88. The revised tool consists of a total of 20 questions, with each question evaluated according to a five-point Likert scale; the total scale ranges from 20 points to 100 points, with higher scores indicating greater job satisfaction.

Empowerment is as a personal characteristic associated with the maintenance of health under stressful conditions. This characteristic was measured by a tool developed by Thomas & Velthouse [16] and validated by Spreitzer [17], with a Cronbach’s alpha of 0.72. The tool consists of a total of 12 questions evaluated according to a five-point Likert scale; the scale ranges from 12 points to 60 points, with higher scores indicating a greater level of empowerment.

Job stress was measured using an abbreviated form of the Korean Occupational Stress Scale (KOSS), developed by Chang et al. [18], with a Cronbach’s alpha of 0.80. The abbreviated KOSS consists of 14 questions regarding the following seven factors: occupational demands, lack of occupational autonomy, relational conflicts, job instability, organizational systems, inadequate compensation, and office culture. Higher scores correspond to greater occupational stress.

A tool developed by Pines et al. [19] and modified and implemented by Kim [20], with a Cronbach’s alpha of 0.91, was used to measure burnout. This tool consists of a total of 20 questions, each of which is answered on a five-point scale. The total scale ranges from 20 points to 100 points, with higher scores indicating more frequent experiences of occupational burnout.

A five-point Likert scale (from 1=not at all to 5=very much) was used to measure job satisfaction, empowerment, job stress, and burnout.

### Statistical analysis

Descriptive analysis was used for the subjects’ general characteristics, and the two-sample *t*-test was used to analyze differences in job satisfaction, empowerment, job stress, and burnout between the nurses and physicians after stratification by institutional type. For the multivariable analysis, we used multiple linear regression to estimate the mean differences in the four index scales (job satisfaction, empowerment, job stress, and burnout) according to sociodemographic and work-related variables after adjusting for potential confounders. SPSS version 12.0 (SPSS Inc., Chicago, IL, USA) was used to organize the data and process the statistics, and p-values<0.05 were considered to indicate statistical significance.

The research protocol for this study was approved by the ethical committee of the Seobuk Hospital (no. 116272-150520-HR-001-01).

## RESULTS

### General characteristics of the research subjects

The general information of the 249 nurses and 57 physicians consisted of the following 11 components: sex, age, marital status, educational level, income, self-rated health, type of hospital, type of job, job position, number of coworkers, years in the TB department, and the average number of days of overtime work per month (Table 1).

Almost of the nurses were female (99.6%), while male physicians (57.9%) outnumbered female physicians (42.1%). The mean ages of the nurses and physicians were 40.6 years and 45.2 years, respectively, indicating that the healthcare professionals in the TB departments analyzed in this study were generally of a relatively advanced age. More subjects were married than unmarried among both the nurses (69.0% married) and physicians (84.2% married). Most nurses held a bachelor’s degree in nursing (88.2%), while less than half of the physicians (41.1%) held a master’s degree (p<0.001). The majority of the nurses (65.9%) were ranked in the middle-income category, while more than half of the physicians (56.0%) were ranked in the high-income category (p<0.001). Levels of self-rated health did not significantly differ between the nurses and physicians.

The majority of nurses (52.6%) worked at public TB hospitals, while the majority of physicians (45.6%) worked at public health centers (p<0.001). The majority of both the nurses (83.1%) and physicians (68.2%) occupied staff positions. The mean number of coworkers for nurses (6.5) was greater than that of physicians (4.0, p<0.001). The number of years in the TB department did not significantly differ between the nurses and physicians. The average number of days of overtime work per month for the nurses (7.0) was greater than that reported by the physicians (2.7, p<0.001).

### Descriptive statistics regarding job satisfaction, empowerment, job stress, and burnout among nurses and physicians

Table 2 shows the mean scores of the four index scales according to sociodemographic and work-related characteristics for nurses and physicians. Both nurses and physicians who reported good self-rated health had higher job satisfaction. Head nurses had the highest level of empowerment, whereas staff physicians had the highest level of empowerment. Physicians who held a bachelor’s degree had higher job stress than their counterparts with higher levels of education. Nurses in the National Medical Center had higher burnout than those working at other institutions.

### Comparison of job satisfaction, empowerment, job stress, and burnout between nurses and physicians

**Figure 1.**
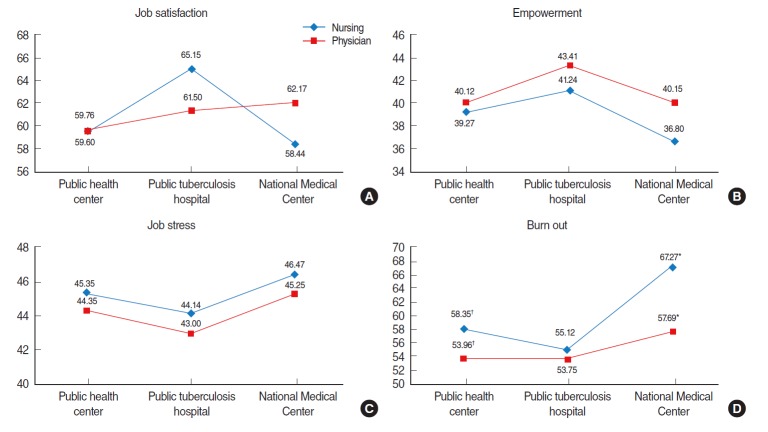
The two-sample t-test was used to analyze differences in job satisfaction, empowerment, job stress, and burnout between nurses and physicians after stratification by institutional type (^†^p<0.1, ^*^p<0.05). (A) Job satisfaction: the mean score of nurses (65.2) was higher than that of physicians (61.5) in the public tuberculosis (TB) hospitals. (B) Empowerment: nurses had the highest score (41.2) in the public TB hospitals. (C) Job stress: nurses had the lowest score (44.1) in the public TB hospitals. (D) Burnout: the mean score of nurses (67.3) was significantly higher than that of physicians (57.7) at the National Medical Center (p<0.05).

In an analysis of job satisfaction, empowerment, job stress, and burnout among the nurses and physicians according to the type of institution, the job satisfaction of the nurses (65.2) was higher than that of the physicians (61.5) in the public TB hospitals, but the score was higher among the physicians (62.2) than the nurses (58.4) at the National Medical Center (Figure 1).

In the public TB hospitals, both the nurses and physicians reported the highest empowerment scores (41.2 and 43.4, respectively) and the lowest job stress scores (44.1 and 43.0, respectively). In all of the institutions (public health centers, public TB hospitals, and the National Medical Center), the levels of job stress and burnout among the nurses were greater than those found among the physicians, but the differences were not statistically significant.

The nurses at the National Medical Center had the highest job stress and burnout, followed consecutively by the nurses in the public health centers and the nurses in the public TB hospitals. The burnout score of the nurses (67.3) was significantly higher than that of the physicians (57.7) at the National Medical Center (p<0.05). In the public health centers, the burnout of the nurses (58.4) was higher than that of the physicians (54.0, p<0.10).

### Predictors of job satisfaction, empowerment, job stress, and burnout among nurses

The multiple linear regression coefficients for each of the four index scales (job satisfaction, empowerment, job stress, and burnout) for the nurses according to sociodemographic and work-related characteristics are presented in Table 3. Job satisfaction was associated with number of coworkers (β=0.514; p<0.05) and self-rated health (β=2.390; p<0.01). Empowerment was associated with a higher job position (β=2.220; p<0.05). Meanwhile, job stress was associated with the average number of days of overtime work per month (β=0.130) and was inversely associated with the number of co-workers (β=-0.227; p<0.05). Lastly, burnout was associated with being unmarried (β=5.981; p<0.01), working at the National Medical Center in comparison with the public health centers (β=4.998; p<0.05), and was inversely associated with job position (β=-3.283; p<0.05), the number of co-workers (β=-0.893; p<0.01), and self-rated health (β=-3.088; p<0.05).

## DISCUSSION

This study found that the job stress and burnout of nurses were higher than those of physicians, and that the level of empowerment of nurses was lower than that of physicians in TB departments in Korean public healthcare institutions.

A previous study showed that registered nurses working in acute-care hospitals in Sweden had lower job satisfaction in larger hospitals [11]. In our study, the job satisfaction of TB management nurses at the National Medical Center, the largest participating hospital, was the lowest among the public healthcare institutions. Also, our study found that the burnout score of the nurses in the TB department of the National Medical Center was significantly higher than that of the physicians. These results may be explained by a heavy workload, as well as by concerns regarding TB infection due to the selection of the National Medical Center for the prioritized hospitalization of severe and highly infectious TB patients, such as MDR-TB patients. Using results from both the tuberculosis skin test and the Quantiferon-TB Gold assay, Lee et al. [21] reported that the yearly rate of TB infection among newly employed nurses in a Korean university hospital was at least 3%. Therefore, stricter preventive strategies against the infection of nurses with TB need to be implemented, such as contact investigations of cases of active TB disease, the installation of isolation rooms in emergency departments, and latent TB infection screenings at the time of hiring.

This study showed that the number of coworkers was associated with job satisfaction among TB management nurses. In a previous study of private hospitals in Korea, the job satisfaction of nurses in institutions with more than two TB control nurses was higher than that of nurses in institutions with only one TB control nurse [23]. In environments with more than two TB management nurses, it was thought that a division of tasks, cooperation, participation in external training during shifts, and effective communication resulted in improved job satisfaction regarding work performance.

Self-rated health was associated with job satisfaction among TB management nurses. A previous study reported a relationship between job satisfaction and health among nurses by showing that increased job satisfaction was related to reduced psychological stress, as reflected by a lower number of CD8+-CD57+ activated T-cells and inflammatory cytokines [4]. Immunological inflammatory changes associated with job satisfaction and psychological distress may be relevant for susceptibility to disease.

This study showed that several sociodemographic and occupational factors were independently associated with the burnout of nurses in TB departments. We found that being unmarried was associated with a higher burnout score among nurses. Previous studies likewise reported that being married was not associated with burnout and was associated with lower emotional exhaustion scores [5,6,8]. Although reports have shown that research subjects who were married or in a cohabitational relationship were prone to family-work conflicts due to greater responsibilities and time demands, resulting in burnout, another study proposed a scenario wherein a supportive partner can assist with the prevention of burnout by alleviating the impact of stress [5].

A previous study showed that lower psychiatric nurse staffing levels and the corresponding lower nurse-to-patient ratios were significantly associated with a higher risk of nurse burnout [9]. In this study, we similarly found that the number of coworkers was inversely associated with burnout among TB management nurses. A previous study found that patients who were cared for by nurses in sufficiently staffed units were more than twice as likely to report a high level of satisfaction regarding their treatment than with other patients, and significantly lower levels of burnout were reported among the nurses responsible for their care [13]. Therefore, TB hospital administrators can use modifiable organizational factors, such as the provision of adequate nurse staffing levels, to focus on improving the quality of patient care.

This study found that the burnout of nurses at the National Medical Center, a large hospital, was significantly higher than that observed among nurses at public health centers. Sellgren et al. [24] reported that unit size was a significant factor in turnover, as a lower turnover rate was reported in those units with 25 or fewer employees than in units staffed by up to 75 employees. In a previous study, overwork in emergency services and lack of job control appeared to represent environmental factors that contributed to significantly higher burnout among community psychiatric nurses than among nurses engaged in other services in Japan [10]. A significant relationship between more favorable work environments and lower levels of nurse burnout were found, and these effects remained strong even after the regression models were adjusted for hospital characteristics such as bed size, teaching status, and technology status [9]. Therefore, changes in the quality of the nursing practice environment, such as the provision of adequate resources, relations with co-workers, and support from supervisors, can improve both job satisfaction for nurses and patient outcomes.

Age was not associated with burnout among nurses in our study. A previous study, however, reported that age was independently and inversely associated with burnout [5]; these associations may be explained by the development of more effective coping strategies by more experienced nurses, but the association may reflect survival bias, as that study was cross-sectional.

Several studies have reported that nurses were the highest-risk group for job stress and burnout among healthcare professionals [21,22]. Moreover, in oncology, intensive, and palliative-care units and in psychiatric services, a higher prevalence of burnout was found among general and hospital nurses who faced chronic or life-threatening diseases [7]; these results may be explained by work characteristics such as high levels of both time pressure and responsibility, in that their role directly influences the patients’ recovery. Existing evidence also suggests that burnout is related to diverse health problems, including depression, drug addiction, suicide, sleep disturbances, and cardiovascular disease [25]; furthermore, nurse burnout can affect patient safety, the quality of patient care, and medical errors [9,26,27]. The prevention of burnout is therefore important for not only the quality of life of individual nurses, but also for ensuring optimal patient care.

Increased requirements for documentation have greatly increased the workload of healthcare professionals, and documentation has become their foremost concern, representing a non-favorable influence on their job satisfaction [26]. As only 57.1% of TB patients were found to be reported to the Korean TB Surveillance System [28], a novel PPM program was established to improve the reporting and management of TB patients. These circumstances have created a considerable amount of administrative tasks for nurses in the TB departments of public health centers, resulting in greater job stress. According to a previous study, raising awareness regarding job satisfaction and burnout is important due to the impact on patient-care quality, poor communication with relatives, and high staff-turnover rates [12]. Therefore, by improving the work environment of nurses, hospitals may discover a relatively affordable strategy for improving the safety and quality of care and increasing the satisfaction of the patients [29].

This study has two major limitations. First, as its design is cross-sectional, it cannot assess causality. Only a few longitudinal studies [4,30] have explored the causal relationship between job satisfaction and burnout in nurses. Future longitudinal studies are therefore necessary to discover the factors that affect these conditions. The external validity of this study regarding job satisfaction, empowerment, job stress, and burnout of TB-treating physicians and nurses was also limited because the subjects of this study all worked at public healthcare institutions and our sample drew from only one tertiary hospital.

According to our results regarding job satisfaction, empowerment, job stress, and burnout among the TB professionals working at the National Medical Center, national TB hospitals, and public health centers in Korea, nurses were more vulnerable to job stress and burnout than physicians; however, in the TB-specialized hospitals alone, the average job satisfaction scores of nurses were higher than those of physicians. Therefore, in Korea, a systematic education program to improve the empowerment of TB management nurses, ensuring sufficient nursing staff levels to reduce the workload of nurses, and obtaining equipment and facilities to prevent TB infections should be considered priorities.

## Figures and Tables

**Table 1.** General characteristics of the respondents

	Nurses	Physician	p-value
Sex			<0.001
Male	1 (0.4)	33 (57.9)	
Female	248 (99.6)	24 (42.1)	
Marital status			0.02
Married	171 (69.0)	48 (84.2)	
Unmarried	77 (31.0)	9 (15.8)	
Education	217 (88.2)		<0.001
Bachelor’s degree	27 (11.0)	18 (32.1)	
Master’s degree	2 (0.8)	23 (41.1)	
Doctoral degree		15 (26.8)	
Income^1^			<0.001
High	25 (11.1)	28 (56.0)	
Moderate	149 (65.9)	17 (34.0)	
Low	52 (23.0)	5 (10.0)	
Hospital type^2^			<0.001
Public health center	102 (41.0)	26 (45.6)	
Public tuberculosis hospital	131 (52.6)	18 (31.6)	
National Medical Center	16 (6.4)	13 (22.8)	
Position			<0.001
Staff nurse/staff physician	196 (83.1)	30 (68.2)	
Charge nurse/senior physician	14 (5.9)	12 (27.3)	
Head nurse/chief physician	26 (11.0)	2 (4.5)	
Age (yr)^3^	40.60± 10.09	45.15± 9.18	0.002
No. of coworkers^3^	6.46±6.55	4.00±4.14	0.001
Years in tuberculosis department^3^	6.40±9.37	6.39±6.24	0.99
Average number of days of overtime work per month^3^	7.03±6.78	2.72±4.67	<0.001
Self-rated health^3^	6.32±1.66	6.70±1.88	0.13

Values are presented as number (%) or mean±standard deviation.

1 Income: high, grade 1-2; moderate, grade 3-5; low, grade 6-10 by Ministry of Government Legislation [14].

2 Public health centers provide primary care, public tuberculosis hospitals provide secondary care, and the National Medical Center provides tertiary care.

3 Two sample t-test.

**Table 2.** Descriptive statistics of the four index scales between nurses and physicians

	n	Nurses				n	Physician			
		Job satisfaction	Empowerment	Job stress	Burnout		Job satisfaction	Empowerment	Job stress	Burnout
Age (yr)										
<39	103	61.0 (8.8)	38.2 (5.1)	45.2 (4.1)	59.9 (10.7)	17	59.9 (7.8)	39.4 (6.7)	45.4 (4.8)	56.7 (12.8)
40-49	81	62.3 (9.3)	40.5 (6.1)	45.4 (4.1)	57.8 (9.6)	16	61.4 (9.7)	42.6 (7.9)	44.9 (5.5)	56.1 (11.2)
≥50	53	65.6 (11.2)	43.7 (6.5)	43.0 (4.2)	51.1 (10.1)	22	61.2 (9.3)	41.6 (7.3)	42.8 (4.2)	52.9 (10.6)
Sex										
Male	1	60.0 (-)	27.0 (-)	50.0 (-)	60.0 (-)	33	61.4 (8.7)	40.7 (8.2)	43.8 (4.5)	54.6 (11.3)
Female	248	62.4 (9.7)	40.2 (6.1)	44.8 (4.2)	57.2 (10.7)	24	59.9 (9.1)	41.8 (5.6)	44.6 (5.3)	55.0 (11.4)
Marital status										
Married	171	62.8 (9.5)	40.8 (6.2)	44.8 (4.3)	55.9 (10.3)	48	60.7 (9.4)	42.0 (6.7)	43.9 (5.1)	54.1 (11.7)
Unmarried	77	61.2 (9.2)	38.4 (5.4)	44.8 (4.0)	60.3 (10.6)	9	61.3 (5.0)	36.9 (8.7)	45.5 (2.6)	58.4 (8.0)
Education										
Bachelor’s degree	217	62.6 (9.5)	40.1 (6.0)	44.9 (4.0)	57.7 (10.4)	18	58.1 (7.7)	37.3 (8.0)	45.2 (5.3)	55.6 (12.3)
Master’s degree	27	59.5 (8.5)	40.4 (5.8)	44.4 (3.9)	55.4 (11.1)	23	58.8 (9.2)	41.7 (5.7)	44.9 (5.1)	58.1 (11.3)
Doctoral degree	2	80.0 (24.0)	50.5 (13.4)	38.0 (17.0)	33.0 (18.4)	15	67.5 (6.2)	44.5 (6.9)	42.0 (2.8)	48.9 (8.3)
Income^3^										
High	25	62.1 (8.0)	40.5 (5.1)	45.9 (4.1)	57.0 (12.1)	28	60.7 (7.9)	42.0 (6.1)	43.3 (4.5)	54.1 (9.5)
Moderate	149	61.8 (9.5)	39.8 (6.1)	45.1 (4.1)	57.5 (10.3)	17	60.9 (9.6)	40.3 (9.0)	45.2 (5.2)	54.6 (12.3)
Low	52	61.8 (9.0)	39.6 (4.6)	44.4 (3.7)	58.3 (9.9)	5	57.6 (4.0)	37.8 (4.0)	47.2 (5.0)	60.2 (8.9)
Position										
Staff nurse/staff physician	196	61.4 (9.2)	39.1 (5.7)	45.3 (3.9)	58.7 (10.4)	30	78.0 (7.1)	57.5 (2.1)	37.0 (<0.01)	34.5 (9.2)
Charge nurse/senior physician	14	60.4 (10.2)	42.5 (4.0)	45.1 (5.1)	51.6 (10.8)	12	61.4 (8.3)	43.7 (5.7)	44.4 (4.1)	56.2 (6.4)
Head nurse/chief physician	26	69.6 (10.9)	46.7 (6.3)	41.2(4.5)	49.8 (9.4)	2	60.8 (8.6)	40.2 (7.2)	43.8 (4.8)	53.8 (12.3)
Self-rated health										
Poor (0-4)	37	56.1 (9.5)	37.5 (5.3)	45.8 (4.7)	66.4 (9.5)	8	53.6 (7.4)	39.4 (7.2)	48.5 (5.0)	65.8 (9.4)
Good (5-10)	211	63.5 (9.2)	40.6 (6.1)	44.6 (4.1)	55.5 (10.1)	49	61.9 (8.5)	41.4 (7.3)	43.4 (4.4)	52.9 (10.5)
Hospital type										
Public health center	102	59.6 (8.6)	39.3 (6.2)	45.4 (3.9)	58.4 (10.8)	26	59.8 (8.7)	40.1 (6.2)	44.4 (5.0)	54.0 (10.6)
Public tuberculosis hospital	131	65.2 (9.7)	41.2 (5.9)	44.1 (4.4)	55.1 (10.1)	18	61.5 (9.9)	43.4 (7.8)	43.0 (4.7)	53.8 (13.4)
National Medical Center	16	58.4 (8.8)	36.8 (5.3)	46.5 (3.8)	67.3 (8.2)	13	62.2 (7.9)	40.2 (8.2)	45.3 (4.8)	57.7 (10.0)

Values are presented as mean (standard deviation).

1 Income: high, grade 1-2; moderate, grade 3-5; low, grade 6-10 by Ministry of Government Legislation [14].

**Table 3.** Adjusted multiple linear regression for the four index scales (job satisfaction, empowerment, job stress, and burnout) among nurses in tuberculosis departments

	n	Job satisfaction	Empowerment	Job stress	Burnout
Age	237	0.004 (0.105)	0.104 (0.067)	-0.041 (0.050)	-0.058 (0.102)
Marriage state	248	-2.416 (1.971)	-2.473 (1.278)^†^	0.288 (0.904)	5.981 (1.857)^**^
Education level	246	-1.119 (1.052)	0.877 (0.675)	-0.175 (0.498)	-0.667 (1.008)
Income	226	-0.033 (0.423)	0.269 (0.271)	-0.247 (0.198)	-0.465 (0.406)
Nurse position	236	1.874 (1.426)	2.220 (0.914)^*^	-0.670 (0.675)	-3.283 (1.376)^*^
No. of coworkers	241	0.514 (0.218)^*^	0.080 (0.141)	-0.227 (0.103)^*^	-0.893 (0.210)^**^
Years in tuberculosis department	226	0.128 (0.112)	-0.038 (0.075)	0.018 (0.055)	-0.028 (0.107)
Average number of days of overtime work a month	217	0.083 (0.109)	0.019 (0.070)	0.130 (0.051)^*^	0.176 (0.105)^†^
Self-rated health	248	2.390 (0.426)^**^	0.348 (0.279)	-0.351 (0.200)^†^	-3.088 (0.411)^**^
Hospital average number of days	249	0.525 (2.363)	1.720 (1.553)	1.530 (1.112)	4.998 (2.269)^*^
Constant		43.972	27.758	48.714	75.402
F		6.872^**^	3.696^**^	1.862^†^	12.486^**^
R^2^		0.323	0.208	0.116	0.463
Durbin-Watson		1.784	1.682	1.898	2.032

Values are presented as beta (standard error). The variables included in the multiple regression analysis were age, marriage state, education level, income, nurse position, number of coworkers, years in the tuberculosis department, days of overtime work in a month, self-rated health, and hospital type. Marriage state: 0, married; 1, unmarried. Education level: 0, bachelor’s degree; 1, master degree; 2, doctoral degree. Nurse position: 0, staff nurse; 1, charge nurse; 2, head nurse. Hospital type: 0, public health center; 1, public tuberculosis hospital; 2, National Medical Center.

† p<0.1,

* p<0.05,

** p<0.01.
